# Adolescents' perception of parental feeding practices: Adaptation and validation of the Comprehensive Feeding Practices Questionnaire for Brazilian adolescents—The CFPQ-*Teen*

**DOI:** 10.1371/journal.pone.0187041

**Published:** 2017-11-16

**Authors:** Ângela Bein Piccoli, Lucas Neiva-Silva, Clarisse Pereira Mosmann, Dara Musher-Eizenman, Lucia C. Pellanda

**Affiliations:** 1 Post Graduation Program in Health Sciences: Cardiology, Instituto de Cardiologia / Fundação Universitária de Cardiologia. Porto Alegre, Brazil; 2 Psychology Graduate Program, Post Graduation Program in Public Health, Universidade Federal do Rio Grande. Rio Grande, Brazil; 3 Psychology Graduate Program, Universidade do Vale do Rio dos Sinos. São Leopoldo, Brazil; 4 Department of Psychology, Bowling Green State University, Bowling Green, United States of America; 5 Universidade Federal de Ciências da Saúde de Porto Alegre. Porto Alegre, Brazil; Universidade Federal de Sao Paulo, BRAZIL

## Abstract

**Background:**

Parental feeding practices may play a key role in dietary habits and nutritional status of adolescents, but research from adolescents’ point of view on this topic is scarce.

**Objective:**

To adapt and validate an instrument of parental feeding practices as perceived by adolescents in a Brazilian setting.

**Methods:**

The Comprehensive Feeding Practices Questionnaire was translated into Portuguese and adapted to be answered by adolescents (ages 12 to 18). Content analysis and FACE validity to assess cultural equivalence was undertaken by experts in the adolescent nutritional and psychological fields. Pilot study was evaluated in 23 adolescents. The final version was administered to 41 students to assess instrument reproducibility (Intraclass Correlation Coefficient). Internal consistency (*Cronbach's Alpha*) and construct validity (Confirmatory Factor Analysis) were assessed in a third sample of 307 adolescents.

**Results:**

Experts and adolescents considered content validity as appropriate. In reproducibility analysis (Intraclass Correlation Coefficient), 10 of the 12 factors were above 0.7. The factors “teaching about nutrition” and “food as reward” obtained values of 0.60 and 0.68, respectively. The *Cronbach's Alpha* of the whole scale was 0.83 and alphas for subscales ranged from 0.52 to 0.85; the factors “teaching about nutrition” and “food as a reward” had the lowest values (0.52). After removing these two factors, the Confirmatory Factor Analysis indicated that the structural model was appropriate. The final scale was made up of 10 factors with 43 questions.

**Conclusions:**

The Comprehensive Feeding Practices Questionnaire-*Teen* demonstrates validity and reliability, and is a suitable tool to evaluate the perceptions of adolescents regarding parental feeding practices.

## Background

Obesity is currently considered one of the most important challenges in public health [1]. Adolescence represents an important period with potentially high risk for the development of obesity and other feeding disorders. Physical and psychosocial complications of obesity may represent significant losses in quality of life. Diseases associated with excessive weight, which previously manifested during adulthood, are now often diagnosed among children and adolescents, including dyslipidemia, cardiovascular disease, sleep apnea, type II diabetes mellitus, and respiratory and orthopedic complications [2–4].

Parents are primarily in charge of the feeding and development of their children’s food habits through behaviors such as incentive, restriction, permission, or passivity in the face of certain foods [5]. Through models that are internalized, children tend to develop eating behaviors like those of their family members [6]. The influence of parental feeding practices on dietary habits and weight status in children and adolescents has become the subject of research in recent years [6–14]. However, although parent-child relationships are bidirectional, the majority of studies focus on the perspective of parents. There is a gap in the literature on instruments to measure the perception of children about their parent’s feeding practices.

This is especially important in this age group, when there is more autonomy and parents may become more permissive. Although adolescents have already acquired greater autonomy in relation to food consumption and lifestyle choices, they are, in many cases, still dependent on routines established by parents for acquiring and preparing food [15]. An instrument that includes food practices perceived by children can be considered a diagnostic tool for health professionals to support their interventions and decisions. For this reason, is important to develop a comprehensive instrument to meet various aspects of nutrition education for adolescents. The Comprehensive Feeding Practices Questionnaire (CFPQ) is a validated scale designed to measure feeding practices for young children (2–8 years old). This instrument was developed by Musher-Eizenman and Holub (2007) and it has been widely used, having been validated in several countries [5, 9, 16, 17] including two recent versions in Brazil [18, 19]. The cross-cultural validation of an instrument of adolescent feeding practices provides a more comprehensive understanding of adolescent health and can contribute to the prevention and treatment of diseases such as obesity which prevalence has been increasing in the world [2].

Two studies have validated instruments to evaluate feeding practices of teenagers’ parents; one as an extension of the Child Feeding Questionnaire (CFQ) for adolescents with an average age of 15 years [20] and another as an extension of CFPQ for adolescents between 10 and 12 years [5]. The factor "parental control" measured by CFQ decreased with the age of the adolescent, which means strategies for controlling diet were less used by parents of adolescents. However, these instruments were adapted to be answered by parents. The lack of valid instruments to measure eating behaviors and parenting styles from the adolescents’ point of view has been a barrier to research in this area, and comparison between studies is challenging [21]. There are differences between perceptions of parents and their offspring about the facts that guide their relationships. These differences are justified by several factors such as personal characteristics, cognitive ability, emotional state and mood, ambient, social context and other concerns. Thus, it is of paramount importance to understand how children and adolescents perceive their parents’ food education, through their practices and styles.

Since the CFPQ provides flexibility for use in multiple configurations and is useful when adapted in different contexts [22], we hypothesized that it could be adapted to be answered by the adolescents themselves. Thus, this study describes the adaptation and validation of the CFPQ in Brazilian adolescents (CFPQ-*Teen*) to assess their perception of parenting feeding practices, verifying its psychometric properties and validity.

## Methods

The study followed the steps for instrument validation recommended in the literature [23,24]: translation and adaptation of the items considering the target population; FACE analyses, (equivalence analysis by expert judges), content validity -; reliability analysis—reproducibility (intra-class correlation coefficient—ICC) and internal consistency (*Cronbach alpha*); and construct validity by confirmatory factor analysis (CFA).

After 7 judges, professionals in the areas of health and education analyzed the instrument as a whole and each item separately; the resulting version was administered to a sample of adolescents for content analysis, verifying that the instrument was appropriate and understandable to the target population. After new adjustments, the final instrument was given to two more samples of adolescents: one to verify the reproducibility of the instrument and the other to check the internal consistency and construct validity. The construct analysis was based on confirmatory factor analysis [25].

This work is part of a study that was approved by the institutional Research Ethics Committee of the Instituto de Cardiologia of Porto Alegre / Brazil with number UP 4648/11. Adolescents were invited to participate, and received an informed consent form for parents or guardians to authorize their participation. The document was signed by the parent (mother or father or guardian) and by the adolescent before inclusion. The questionnaires were completed in the schools, in groups of students, according to the schedule authorized by the schools.

### Participants

Three different samples of adolescents between 12 and 18 years, enrolled in 6 schools of the city of Porto Alegre, south Brazil, were included. Four public schools and two private schools were chosen from a list of schools provided by the local Education authority, based on geographic distribution. The schools were located in different neighborhoods, comprising all the main town’s regions. No school refused participation.

Sample 1: This sample included 23 adolescents (61% female) between 12 and 18 years, with a mean age of 15.4 (±1.74) years who were invited to participate in the study for semantic analysis of content. This was a convenience sample, composed of sons and daughters of adults who were approached about the possible participation of their children in a pilot study for the development of the instrument. The adults were students in a school located in the hospital—Instituto de Cardiologia of Porto Alegre–that provides adult-education courses in nursing and nutrition. The adolescents came from different regions of the city.

Sample 2: For the analysis of reproducibility and reliability, we randomly selected 148 adolescents (48% female) from three public schools, with mean age 14.7 (±1.02). Of these, 55 (37.1%) responded to the scale a second time after 2–4 weeks; and 41 provided complete data. Two factors contributed to the low adherence to the second application: absence of the students who answered the first time in two of the schools and the interruption of the classes in the third school. The sample of 41 students was considered satisfactory for the objective of reproducibility analysis.

Sample 3: Two public and two private schools were involved. For the internal consistency (reliability analysis) and construct validity, the instrument was administered to 307 adolescents (58% female) between 12 and 18 years and 58% from public schools, which represent families with low socioeconomic status and lower purchasing power. Of the 307 adolescents, 63.2% were living with both parents, 79.8% reported the mother as the main caregiver (Table 1).

**Table 1:** Characteristics of participants and their families.

	Sample 1 (N = 23)	Sample 2 (N = 41)	Sample 3 (N = 307)
Adolescent’s Characteristics	N (%)	N (%)	N (%)
**Gender**			
Male	9 (49.0)	21 (52.0)	129 (42.0)
Female	14 (61.0)	20 (48.0)	178 (58.0)
**Age**			
12–13	5 (21.7)	12(29.3)	77 (25.1)
14–15	6 (26.1)	28 (68.3)	114 (37.1)
16–18	12 (52.2)	1 (2.4)	116 (37.8)
**Schools**			
Public	17 (73.9)	41 (100)	175 (57.0)
Private	6 (26. 1)	-	132 (43.0)
**Education**			
Elementary school	12 (52. 2)	41 (100)	129 (42.0)
High school	11(47.8)	-	178 (58.0)
**Family**			
Lives with both parents	-	-	194 (63.2)
Lives with remarried parents	-	-	75 (24.4)
Lives with only one of the parents (separated or death)	-	-	37(12.1)
No answer	-	-	1 (0.3)
**Father’s Education Level**			
No schooling and incomplete elementary school	-	-	58 (18.9)
Complete Elementary School	-	-	43(14.0)
Complete High School	-	-	90 (29.3)
Complete College Education	-	-	99 (32.4)
No answer			17 (5.5)
**Mother’s Education Level**			
No schooling and incomplete elementary school	-	-	48(15.6)
Complete Elementary School	-	-	35 (11.4)
Complete High School	-	-	106 (34.5)
Complete College Education	-	-	112 (36.6)
No answer			6 (1.9)

The CFPQ has 12 factors, with 49 questions scored on a *Likert* 5-point scale to evaluate different parental feeding practices related to children’s nutrition: child control, emotional regulation, encouraging balanced and varied diet, environment, parental modeling, parental monitoring, food as reward, restriction for weight control, restriction for health, pressure to eat, involvement and teaching about nutrition [22].

### Procedures

**Fig 1:**
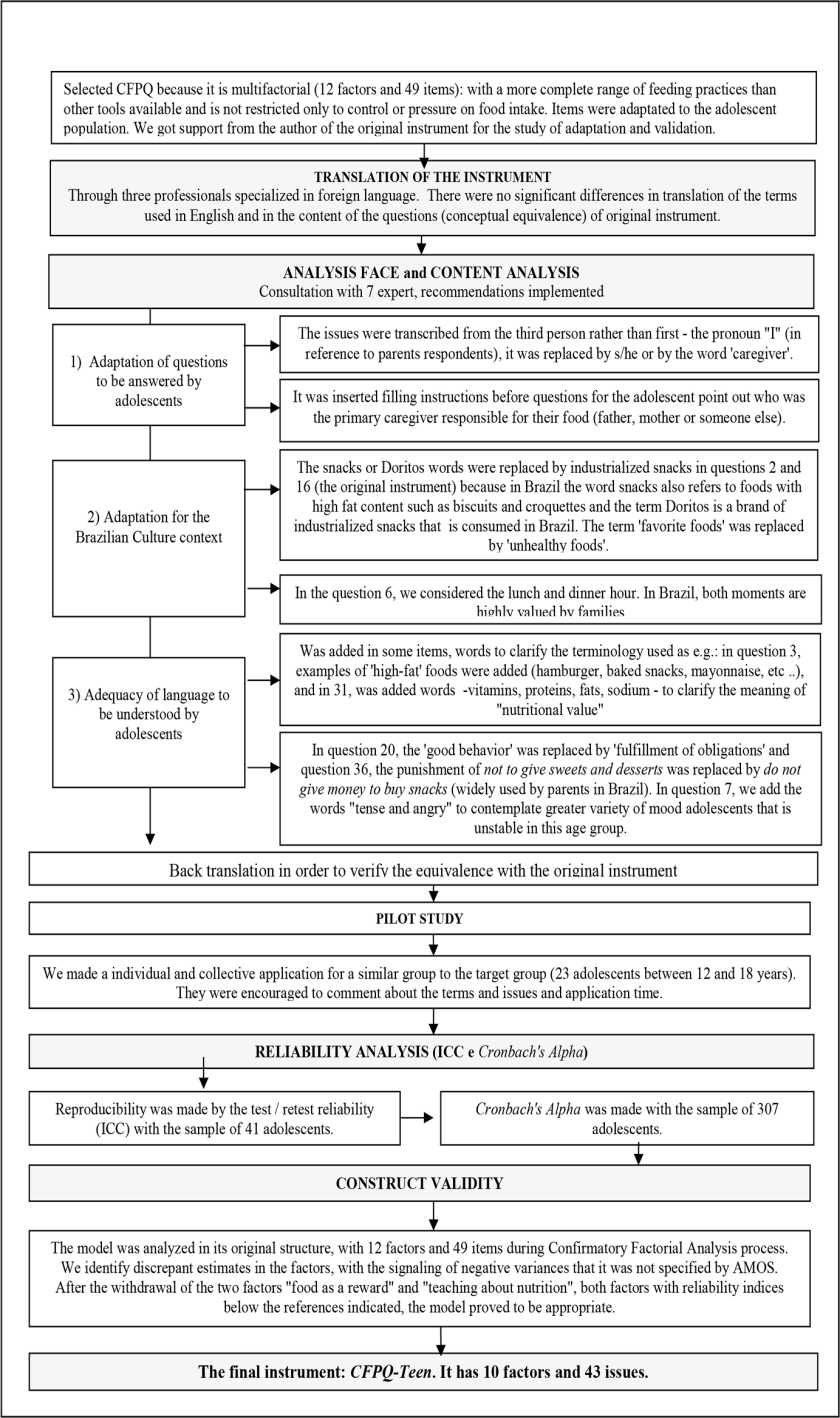
Stages of the process of adaptation and validation of CFPQ for adolescents in southern Brazil.

The process of adaptation and validation CFPQ for adolescents is summarized in Fig 1 and described below in steps.

Three professionals with expert knowledge of English and Portuguese translated the instrument from English into Portuguese separately. Two Brazilian professionals had specialization in foreign languages—Portuguese and English—and one was a Brazilian resident in the United States for over 30 years working with translations of English documents into Portuguese. There were no divergent translations regarding the understanding of the terms used in English or in the content of the questions (conceptual equivalence). Since the instrument would be answered by teenagers and not by parents, the process of back translation was adapted since pronouns were changed ('I' would be replaced by he/ she), and some terms were modified for age group suitability (e.g., 'good behavior' was replaced by 'fulfillment of obligations') thus rendering an exact back translation impossible (supplementary material). We adapted the instrument using appropriate language to this age group and all questions were written in the third person. Instead of the pronoun "I" implicit in the questions that were answered by parents about their own practices, the pronoun was replaced by he/she in reference to parents. For example, the original CFPQ item *“I involve my child in planning family meals”* was adapted to assess the perception of adolescents, resulting in “This caregiver asks my opinion on planning meals and menus for the family” (in Portuguese: *“Este(a) cuidador(a) pede minha opinião no planejamento das refeições e cardápios da família”)*. Special attention was given to the instructions for completing the questionnaire, in which the adolescent would respond by thinking about the caregiver who is most occupied with their food (e.g., mother, father, stepfather, stepmother, or grandparent).

### FACE validity

After the translation and adaptation process and before the pilot application, FACE validity was performed. This analysis is qualitative, involving expert judges, who analyze the representativeness of the items in relation to the content and relevance of the objectives to be measured [26]. Seven judges who were considered specialists on the matter evaluated the instrument. The judges were professionals in the areas of health and education: two physicians (one adolescent psychiatrist and one specialist in obesity and eating disorders), three psychologists (with specializations in the area of family, adolescence, and obesity) one expert in education, and one nutritionist. The instrument was sent by email to each expert, who performed an independent analysis without any input from the other judges. The documents were returned by e-mail to the authors and the first adjustments were made.

### Content validity (Pilot study)

After performing the recommended adjustments, 23 adolescents (Sample 1) participated in a pilot study to evaluate the “content analyses” of the instrument to verify the adequacy of language, the ability to identify understanding of the questions and the relevance of the item. The adolescents were free to express themselves while completing the questionnaire if they did not understand a word or a question. After the questionnaire, they were asked to discuss their experience in answering the instrument, if they agreed or disagreed with any particular item and if they considered the instrument difficult, tiresome, or boring. The instrument was considered by five teenagers as too long. Since the average time to complete was about 20 minutes, this point was valued as important for future decisions on the qualitative aspect of the application and to understand adolescent personality characteristics.

### Reliability analyses

In the next step, reliability was evaluated, by evaluating the reproducibility and consistency of measurement [27]. First, focusing on test / re-test reliability [28], participants (Sample 2) completed the instrument on two occasions, with an interval of 2 to 4 weeks, according to the schedule provided by the schools.

### Construct validity

In the final step, to evaluate the internal consistency and the construct validity, the *Cronbach’s Alpha* (α) and Confirmatory Factor Analysis (CFA) was performed in Sample 3 to ensure the quality of the measurement [29].

### Statistical analysis

Data were analyzed using SPSS, version 19. The distribution of scores on each factor was assessed by calculating the average of the items composing each factor. To validate the reliability of CFPQ-Teen, we used the test-retest reproducibility method, using the Interclass Correlation Coefficient (ICC) and *Cronbach's Alpha* (α) to measure the internal consistency of the scale once its items define a multifactor structure [30]. For construct validity, we established as a fundamental analyses, the comparison of the results of CFPQ-Teen with the results of the original questionnaire and other validation studies of the same instrument. So, we assumed the theory which underlay the original scale about the association between overt behavior (variables) and factors (latent variables) and we sought to confirm the degree of fit of the observed data with the theory considered. In order to accomplish this, we used Confirmatory Factor Analysis (CFA) with structural equation modeling carried out with the help of AMOS software, version 16.0. Several goodness of fit measures assessed empirical support for construct validity [i.e., χ² (Chi-Square), Root Mean Square Error of Approximation (RMSEA), and incremental fit statistics including Normed Fit Index (NFI), the Comparative Fit Index (CFI) and the Tucker-Lewis index (TLI)] [31]. Finally, measures of parsimonious fit relate the fitness of the model to the number of coefficients required to achieve this level of adjustment (Parsimonious Normed Fit Index—PNFI). The following criteria were used: 1) The ratio of chi-square relative to the degrees of freedom (χ²/g.l. or CMIN/DF) between 1.0 and 3.0 and less rigorous, accepted upper limit of 5.0; 2) RMSEA (90% CI): recommended values below 0.08 (AMOS) or below 0.06; 3) The Comparative Fit Index CFI; 4) NFI; 5) TLI; 6) Incremental fit Index (IFI) whose general recommendation points to indexes above 0.90; 7) The Parsimonious Normed Fit Index (PNFI) greater than 0.60 indicates good parsimony, however, some authors consider the value 0.50 as good [31]. Remember that the established cutoff points are arbitrary and the model is considered appropriate when at least three indices are within the parameters considered.

## Results

### Content validity

In general, the judges and adolescents considered the instrument appropriate for the proposed age group. The judges assessed the content and wording of the instrument and the relevance of the questions in the Brazilian context and considered the responses in the *Likert* scale format to answer the specific needs of adolescent age group. Some suggestions were made. According to the original scale of Musher-Eizenman & Holub (2007), the response-formats are 'never, rarely, sometimes, mostly, always' (items 1–13) and 'disagree, slightly disagree, neutral, slightly agree, agree' (14–49). During the content analysis, the judges proposed small changes to be more appropriate to the adolescent comprehension. The adolescent version become 'never, almost never, sometimes, often, always' and 'totally disagree, partly disagree, neither agree or disagree, partly agree, totally agree'.

One judge suggested using examples to clarify about “fatty foods” in question 3 (How often does this caregiver control the quantity of fatty food that you eat?), adding the words "hamburger, snacks, mayonnaise, etc" since not all high calorie and unhealthy food are recognized by adolescents as fatty foods. In question number 7 (When you are irritable, does this caregiver offer you something to eat or drink?), the judges suggested that the state of angry mood could be accompanied by other forms of expressing irritation, and thus the terms “tense” and “agitated” were added (When you are agitated, tense or irritable, does this caregiver offer you something to eat or drink?). In question 36, the original statement revealed a punishment for bad behavior restricting access to sweets or desserts. For the adolescent age group, the judges considered interesting to add others forms of punishment, e.g. restricting cash for snacks, widely used in Brazilian culture. Thus, the item was changed from "The caregiver restricts sweets and dessert in response to bad behavior" for "When I behave badly, he / she does not give me money for snacks".

The adolescents suggested replacing the word “restricts” for “limits” (question 34, the original version) and underlining certain words in the instructions to provide emphasis. The opinion of relevance of the questions varied according to age. Older teenagers considered some questions as ‘inadequate for their age’, such as question 36, already mentioned. However, since the majority of adolescents considered all the questions relevant, all questions were retained to establish a basis for comparison in the analysis of psychometric properties. In general, adolescents considered the tool easy to understand, taking an average of 15 minutes to complete the scale individually and 25 minutes when used in a group.

### Analysis of reliability

Table 2 presents the reliability analyses (*Cronbach’s Alphas* and ICC). In the test/re-test analysis, 10 of the 12 factors were above the value of 0.7, allowing estimates of reproducibility in most factors except “teaching about nutrition” and “food as a reward”, which obtained the values 0.60 and 0.68 respectively. The alpha values were between 0.52 to 0.85. The scale total alpha was 0.83.

**Table 2:** Interclass Correlation Coefficient (ICC) and internal consistency of factors according to Cronbach’s Alpha.

Factors of Feeding Practices	Items	ICC N = 41	*Cronbach’s Alpha* n = 307
Adolescent Control	5, 6, 10, 11, 12	0.91	0.67
Emotional regulation	7,8,9	0.86	0.77
Encouraging balance and variety	13, 24, 26, 38	0.81	0.66
Environment	14,16,22,37	0.86	0.64
Involvement	15,20,32	0.87	0.56
Parental modeling	44, 46, 47, 48	0.83	0.82
Monitoring	1, 2, 3, 4	0.79	0.85
Pressure to eat	17,30,39,49	0.86	0.56
Restriction for Health	21,28,40,43	0.81	0.62
Restriction for Weight Control	18,27,29,33,34, 35,41,45	0.90	0.83

Note: The numbering of items of each factor is in accordance with the initial scale, considering the twelve factors.

There were differences in alpha coefficients when some items were removed from the scale. Removing items 36 (‘When I behave badly, he / she does not give me money for snacks’) and 42 (‘This person says what I have and what I do not have to eat without much explanation’) significantly increased the alpha values from 0.52 to 0.59 and 0.52 to 0.60 respectively. However, in factor analysis, at least three variables per factor are recommended [5] and, consequently, the removal of these items from the two factors (“food as reward” and “teaching about nutrition”) was not performed at this stage of analysis. Results from the construct validity analyses were considered to support the subsequent decision to remove these factors (Table 3).

**Table 3:** Resulting indices of confirmatory factor analysis of Comprehensive Feeding Practices Questionnaire for adolescents (CFPQ- Teen)^a^.

Measures of Goodness of Fit^b^	Level of Fit recommended	Value CFPQ-*Teen*
Measures of Absolute Fit		
**CMIN/DF (χ²/df)**	< 3.0	**1.981**
**RMSEA**	< 0.06	**0.057**
Measures of Incremental Fit		
**NFI (Δ1)**	> 0.80	0.702
**TLI**	> 0.95	0.792
**IFI**	≥ 0.90	0.826
**CFI**	> 0.90	0.821
Measures of Parsimonious Fit		
**PNFI**	> 0.60	**0.605**

Note.

^a^ Results obtained after the removal of factors “food as reward” and “teaching about nutrition”;

^b^Measures of Goodness of Fit: CMIN/DF = normal chi-scare; NFI = Normed Fit Index or Delta 1; TLI = Tucker-Lewis Index; IFI = Incremental Fit Index; CFI = Comparative Fit Index; PNFI = Parsimonious Normed Fit Index; RMSEA = Root Mean Square Error of Approximation

### Construct validity

**Fig 2:**
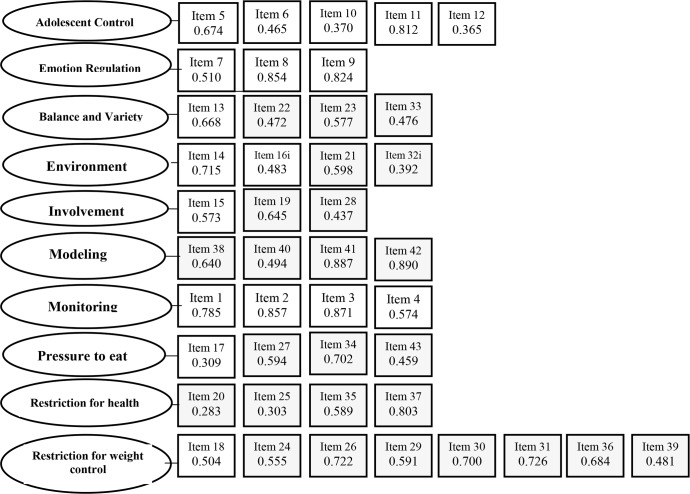
Factor loading for each item. Values in boxes give factor loadings from Confirmatory Factor Analysis (by AMOS). The questions are numbered according to the order of application in the final scale and the gray boxes refer to items that have changed their numbering in relation to the original instrument.

Initially, the structural model was tested with the 12 factors and 49 items in which its discrepant estimates were found in the factors and signaling negative variances not specified by AMOS. The factors ‘food as reward’ and ‘teaching about nutrition’ showed low internal consistency values, both with 0.52. These data indicated the decision to remove both factors. After removing the two factors, the model was considered appropriate, according to the results shown in Table 3, compared to the recommended values for these indices [31]. Factor loadings of the items ranged from 0.283 to 0.890, with the majority (86%) being above 0.459 (Fig 2).

The final protocol, after the validation process, contained 43 questions in grouped 10 factors: ‘adolescent control’ (renamed from child control), ‘emotional regulation’, ‘encouraging balanced and varied diet’, ‘environment’, ‘parental modeling’, ‘parental monitoring’, ‘restriction for weight control’, ‘restriction for health’, ‘pressure to eat’, and ‘involvement’. The factors are more fully described in S1 File.

## Discussion

In this validation study, we concluded that the CFPQ instrument could be adapted for Brazilian adolescents, retaining its reliability and validity. The choice of *CFPQ* as a reference tool for the design of an evaluation instrument and analysis of parental feeding practices of teenagers was based on the similarity between the food culture of North and South America. They have a certain conceptual equivalence of practices parental food, especially in southern Brazil [32–34]. Another reason for choosing the CFPQ to evaluate parental feeding practices was that parental attitudes expressed in the instrument are mostly also directed at teenagers. The different feeding practices adopted by parents were identified through a multi-dimensional structure such as encouraging the practice of eating well and at regular intervals, using food as an educational strategy (rewards or autonomy, for example) the concern in the selection and preparation of food; focusing on specific issues related to weight and health; parents are examples of good practice, monitoring, etc. In literature for adolescent age group it was found that such practices reinforced the initial maintenance all factors in CFPQ for use with adolescents [35].

Two studies have validated instruments to evaluate feeding practices of teenagers’ parents; one as extension of the CFQ for adolescents with an average age of 15 years [20] and another as an extension of CFPQ for adolescents between 10 and 12 years [6]. In the first study, the factor "parental control" measured by CFQ decreased with the age of the adolescent, which means strategies for controlling diet were less used by parents of adolescents. The second study eliminated two factors (food as a reward and as a regulator of emotions) from the initial research with parents, who considered these factors irrelevant.

However, these instruments were adapted to be answered by parents. The lack of valid instruments to measure eating behaviors and parenting styles from the adolescents’ point of view has been a barrier, and comparison between studies is challenging [36]. There are differences between perceptions of parents and of their offspring about the facts that guide their relationships. These differences are justified by several factors such as personal characteristics, cognitive ability, emotional state and mood, ambient, social context and other concerns, involved in the process of communication between parents and children [37]. Thus, it is of paramount importance to understand how children and adolescents perceive and internalize their parents’ food education, practices, and styles.

Parenting practices adapt to suit different needs and demands according to the growth and maturity of the child. This is also true in the domain of food practices. Furthermore, the degree of maturity and emotional balance also varies widely among adolescents, highlighting the dynamic nature and bidirectional interactions between parents and children [38]. On the other hand, parents’ concerns regarding the feeding of children is that they develop and grow healthy, but when the children grow up, parents often express concern when disease or an unhealthy nutritional status or even a sedentary lifestyle are established in the adolescent. Parents have difficulty in recognizing that the family atmosphere and adopted feeding practices may be influencing the nutritional status and sedentary lifestyle [39]. When disease is established (obesity, eating disorders, dyslipidemia, high cholesterol or high blood pressure, for example), the healthy eating habits play a role of *healthcare*, overlapping the preventive role [40]. Thus, it is critical to understand how adolescents perceive parental recommendations for nutrition, and the validation of an instrument that identifies the perception of adolescents in relation to their food education is a very important step in this process.

The Adolescence range is wide, and younger adolescents are still emotionally and economically dependent on their parents. They still need them as role models, guides and disciplinarians of dietary habits. Another aspect considered refers to the maturity and emotional balance in this period, characterized by being of great instability when the adolescent is more susceptible to outside influences [38].

The cognitive, social, and emotional development of the child influences the selection of feeding strategies by parents. Thus, tests that assess dietary practices developed for parents of young children do not necessarily apply to parents of teenagers. For example, some instruments (e.g., Caregivers Feeding Style Questionnaire, Hughes et al., 2005) that assess feeding style, ask parents if they tell the child that “milk is good for your health because it will make you strong”, or if they encourage children to eat by “making smiley faces on the pancakes”. Items like this are intended to focus on younger children and do not address the adolescent age group [41].

For this reason, the CFPQ with its scope and specificity meets many aspects of food education for adolescents. The reliability and validity analyses done here showed compatible results with those of the original [22] and related studies [5,9,16]. However, regarding stability, the factors "teaching about nutrition" and "food as reward" presented rates slightly lower than expected.

As for internal consistency, generally speaking, an instrument is classified as having adequate reliability when α is at least 0.70 [16]. However, in some scenarios of research in the social sciences an α of 0.60 is considered acceptable provided that the results obtained with this instrument are interpreted with caution [36]. The high consistency in the presence of multidimensionality indicates that the items that make up the different dimensions of a measure are strongly correlated, despite the fact that the dimensions are less correlated than the items that compose them. CFPQ-*Teen* demonstrated acceptable reliability (α> 0.60) in eight of the twelve factors (67%). The factors with a value of < 0.60 were: “environment”, “food as reward”, “involvement”, “pressure to eat” and “teaching about nutrition”. These indices, however were compared with other studies of validation of CFPQ and indicated, in general, a decrease compared to the original study [22] (with children 2–8 years). However, in relation to studies for parents of children aged 10 to 12 years [5], the alpha was higher in factors: “adolescent control”, “environment”, “parental modeling”, and “teaching about nutrition”, and showed similarities in the factors “encouraging balance and variety”, “restriction for weight control”, and “parental monitoring.” We observed that most of the factors with low *alphas* were those that comprised only 3 items. Taken together, the *alpha* values found in this study were deemed acceptable.

The internal consistency analyses identified some items—7, 17, 22, 36 and 42—that could be removed to improve internal consistency, but were kept considering the statistical and conceptual relevance of the question. Question 7, for example (“When you are agitated, tense or irritated, does the caregiver offer you something to eat or drink?”), from the factor “food for emotional regulation” was not removed for two reasons: 1) because the factor *alpha* value was adequate (0.77) and the difference was only 0.03; and 2) the question belonged to a factor with only three items which would require the removal of the factor from the scale. However, we do not exclude the possibility of further analysis of the item and factor for this age group. The referenced item refers to three different expressions for “irritation” in a single question, which may have caused increased variability of responses by the adolescent. Similarly, the removal of Item 17 (“I must eat all the food on my plate”) would increase the value of the *alpha* by 0.04. However, it is an item that deserves attention, since, being linked to pressure for eating, it is common for parents to require their children, even teenagers, not to waste food.

We tested if the theoretical factor structure fits the observed data with CFA. Additionally, the Confirmatory Factor Analysis allows us to test the adjustment factor relating to competing models. This type of analysis is of great value in the review process and refinement of psychological instruments and their factor structures. In this study, the factor analysis results were a deciding point for removing the factors “teaching about nutrition” and “food as a reward”. It is not clear whether this reflects that these constructs are not relevant for the adolescent sample or whether the included items did not adequately tap these constructs. It is possible that the construct "teaching about nutrition" factor makes more sense when it is reported by the parent than when it is answered by adolescents. It would be useful for research to explore this further. This may be an aspect to be re-evaluated in future research through a dimension of ‘communication between parents and children’ instead of teaching strategies.

Previous research with younger children has indicated that controlling feeding practices may be especially problematic in the development of healthy eating and weight outcomes [42]. Given the expectation for increasing autonomy in adolescence, these practices might be even more worthy of attention in this population. For example, “restriction for health and weight” and “pressure to eat” may be possible indicators of pathology, as noted later, when these practices were associated with overweight and obesity.

One limitation of this study is that its findings are based on a cross-sectional survey with a sample restricted to adolescents in a city in southern Brazil. Studies that investigate evidence of validity in other contexts are essential so the CFPQ-*Teen* can have its validation extended in other contexts. Research on parental feeding practices for adolescents is a challenge because, at this age, teens have more autonomy and parents may become more permissive and support this autonomy. For example, one adolescent in the sample described how she became a vegan and thus no longer delegated food preparation to her parents. In this case, factors such as ‘environment’, ‘encouraging the intake of different foods with balance’ or ‘involvement’ may take on different meaning as adolescents establish an independent identity concerning food.

Other critical clinical conditions, such as diabetes or eating disorders, increase the concerns of parents and feeding practices focused primarily on the health and preservation of life. In this regard, although the CFPQ*-Teen* has a dimension of 'food restriction for health', the items do not address the care practices aimed at the treatment of specific clinical conditions. Further validation studies are suggested for these specific groups.

## Conclusions

The CFPQ-*Teen* demonstrates validity and reliability, and is a suitable tool to evaluate the perceptions of adolescents regarding parental feeding practices. Urban Brazilian adolescents share many similarities with other adolescents, especially of other developing countries, and the CFPQ-*Teen* is a promising tool for future research exploring the perceptions of adolescents about the educational feeding practices of parents and their effects on health. Adolescents are increasingly overweight worldwide, and the study of the family context is of paramount importance.

## Supporting information

S1 File Comprehensive Feeding Practice Questionnaire for adolescents (CFPQ-*Teen*). (DOC)

S2 File Databases. (SAV)
