# Supplementary material for: Adolescents' perception of parental feeding practices: Adaptation and validation of the Comprehensive Feeding Practices Questionnaire for Brazilian adolescents—The CFPQ-Teen
Source: PLoS One. 2017 Nov 16;12(11):e0187041. doi: 10.1371/journal.pone.0187041 (PMC5690605; doi:10.1371/journal.pone.0187041)
Supplement: S1 File — (DOC) [file pone.0187041.s001.doc]

**Comprehensive Feeding Practice Questionnaire for adolescents (CFPQ-*Teen*)**

Below, the **Comprehensive Feeding Practice Questionnaire - CFPQ for adolescents - 12 to 18 years old** **(CFPQ-*Teen*)** - with 10 factors with a brief operational definition and the 43 relevant questions. The questions were adapted to be answered by adolescents about the feeding practices adopted by their parents. The questions are organized by dimensions and they are numbered according to the order of application in the final scale. The questions are subject to a *Likert* 5-point scale with the response options for questions 1-13 being “never, almost never, sometimes, often, always” and questions 14-43 use a 5-point scale with different anchors: “I totally disagree, partly disagree, neither agree or disagree, partly agree, totally agree”. Items 16 and 32 are reverse scored.

Nomenclature was also maintained in 9 factors and the factor “Child Control” was renamed “Adolescent Control”. Each factor includes 3-8 questions:

**Adolescent Control:** **Assesses the frequency at which parents are more permissive regarding food behavior and habits in adolescents.**

5. This caregiver allows you to eat whatever you want?

6. Can you choose the items you want of what is served at lunch or dinner, leaving aside what you do not like, without interference from the caregiver?

10. When you do not like what is served for eating, does your caregiver cook something else for you?

11. Does this caregiver allow you to have snacks whenever you want?

12. Does this person allow you to leave the table when you are satisfied, even if others have not finished eating?

**Emotion Regulation:** **Assesses how adolescents perceive their parents use food to regulate unsatisfactory mood or emotions.**

7. When you are agitated, tense or irritable, does this caregiver offer you something to eat or drink?

8. When you are bored, does this person offer you something to eat or drink even when aware that you are not hungry or thirsty?

9. When you are sad, does this caregiver offer you something to eat or drink even when aware that you are not hungry or thirsty?

**Encouraging balance and variety:** **evaluates how adolescents perceive their parents as the ones who encourage the intake of various** **foods with nutritional balance.**

13. Are you encouraged to eat healthy food items instead unhealthy ones?

**22**. I am encouraged to try new food.

**23**. This person tells me that healthy food tastes good.

**33**. I am encouraged to eat a variety of food

**Environment:** **measures the availability of healthy foods at home.**

14. Most of the food at home is healthy.

16. At home, there is a lot of snacks (potato chips, salty popcorn, etc.).**R**

**21**. During meal times at home, there are several healthy food items available for me to eat.

**32**. At home, there is a lot of sweets (ice cream, cakes, pies, candies/preserves, desserts, goodies).**R**

**Involvement:** **evaluates the encouragement given by parents to their adolescent children to participate in planning and preparing meals.**

15. This caregiver asks my opinion in the planning of family meals and menus.

**19**. This person allows me to help in the preparation of family meals.

**28**. This person encourages me to participate in grocery shopping

**Modeling**: **assesses how adolescents perceive their parents as a model or reference for them regarding healthy food habits.**

**38**. This caregiver eats healthy food to give me an example of healthy eating habits.

**40.** Even when it is not the caregiver's preferred food, s/he often eats it because s/he finds it important to give me her/his example.

**41**. S/he tries to show enthusiasm regarding healthy food.

**42**. S/he shows me how much s/he enjoys eating healthy food.

**Monitoring:** **assesses the adolescents' perception regarding the frequency with which parents monitor the consumption of unhealthy foods.**

1. How often does this caregiver keep track of the quantity of sweets (or ice cream, cakes, pies, chocolates, candies) that you eat?

2. How often does this caregiver keep track of the quantity of industrialized snacks (potato chips, munchies, cheese pastries, etc.) that you eat?

3. How often does this caregiver keep track of the quantity of fatty foods (hamburgers, snacks, mayonnaise, etc.) that you eat?

4. How often does s/he keep track of the quantity of sweet drinks (soda/soft drinks, juices) that you drink?

**Pressure to eat:** **measures the perception of adolescents as to how parents pressure for greater food intake during meals.**

17. I must eat all of the food on my plate.

**27**. If I say "I'm not hungry", my caregivers insist that I eat anyway.

**34**. If I eat only a small helping, I am encouraged to eat more.

**43.** When I finished eating, my caregivers try to offer me a little bit more.

**Restriction for health:** **evaluates the perception of adolescents regarding parental controls to limit the consumption of less healthy foods or sweets.**

**20**. I could eat too much of my favorite food if there were not any control over my eating.

**25**. I would eat too much "junk food" if there were not any control over my eating.

**35**. S/he controls me not to eat a lot unhealthy food.

**37**. This person controls me not eat too many sweets, small meals, snacks.

**Restriction for weight control:** **assesses the adolescents' perception of parental control over food intake to decrease or maintain their weight.**

18. This caregiver needs to be sure that I do not eat fatty foods.

**24**. This person encourages me to eat less food so that I won’t get fat.

**26**. This person helps me controlling the quantity of food that I serve myself at each meal in order to control my weight.

**29**. If I eat more than normally at one meal, this person reduces the quantity of food at the next meal.

**30**. This caregiver limits the foods that might make me fat .

**31**. S/he believes that I should not eat certain foods so that I do not gain weight.

**36**. I am monitored so that I do not eat between meals in order to not get fat.

**39**. This caregiver -forces me to restrict my diet in order to control my weight.

The general instructions of the scale, also in Portuguese, are: **“**Parents or other relatives have different concerns over the eating of their children and may have different attitudes and behaviors in that regard. Please answer the questions below thinking of the behavior of your parents or some other person who monitors the way you eat. There are no right or wrong questions. Please try to be the most honest you can in your answers.”

As you respond, think of only one person, of the one person that mostly takes responsibility for your eating: only your mother, only your father or only someone else. The person you choose will be referred to as caregiver.

I will answer thinking of: ( ) my mother Age: ..............

( ) my father Age: ..............

( ) somebody else Who? ....................... Age:............

For questions 1-13, the instructions are: “Answer the questions below checking with an X how often you notice that the following situations happen regarding the caregiver that monitors your eating. Select only one option for each question.”

For statements 14-43, the instructions are: “Now, regarding the affirmations below, mark with an X the degree to which you agree (1, 2, 3, 4, 5) regarding how much each sentence describes what really happens in your home, with nº 1 being when you TOTALLY DISAGREE and nº 5 when you TOTALLY AGREE.”

“Select only one answer for each sentence.”
